# Cell Adhesion and Shape Regulate TGF-Beta1-Induced Epithelial-Myofibroblast Transition via MRTF-A Signaling

**DOI:** 10.1371/journal.pone.0083188

**Published:** 2013-12-10

**Authors:** Joseph W. O’Connor, Esther W. Gomez

**Affiliations:** 1 Department of Chemical Engineering, The Pennsylvania State University, University Park, Pennsylvania, United States of America; 2 Department of Bioengineering, The Pennsylvania State University, University Park, Pennsylvania, United States of America; University of California, San Diego, United States of America

## Abstract

Myofibroblasts, specialized cells that play important roles in wound healing and fibrosis, can develop from epithelial cells through an epithelial-mesenchymal transition (EMT). During EMT, epithelial cells detach from neighboring cells and acquire an elongated, mesenchymal-like morphology. These phenotypic changes are accompanied by changes in gene expression patterns including upregulation of a variety of cytoskeletal associated proteins which contribute to the ability of myofibroblasts to exert large contractile forces. Here, the relationship between cell shape and cytoskeletal tension and the expression of cytoskeletal proteins in transforming growth factor (TGF)-β1-induced EMT is determined. We find that culturing cells in conditions which permit cell spreading and increased contractility promotes the increased expression of myofibroblast markers and cytoskeletal associated proteins. In contrast, blocking cell spreading prevents transdifferentiation to the myofibroblast phenotype. Furthermore, we find that cell shape regulates the expression of cytoskeletal proteins by controlling the subcellular localization of myocardin related transcription factor (MRTF)-A. Pharmacological inhibition of cytoskeletal tension or MRTF-A signaling blocks the acquisition of a myofibroblast phenotype in spread cells while overexpression of MRTF-A promotes the expression of cytoskeletal proteins for all cell shapes. These data suggest that cell shape is a critical determinant of myofibroblast development from epithelial cells.

## Introduction

Myofibroblasts, specialized cells within the body that exert large contractile forces, mediate wound healing and upon aberrant activation contribute to the development of fibrosis and cancer [1–4]. The contractility of these cells is governed by specialized matrix adhesions [5] and distinct cytoskeletal organization characterized by contractile bundles of actin and myosin [6]. A hallmark of the myofibroblast phenotype is the expression of alpha smooth muscle actin (αSMA), a cytoskeletal protein which promotes increased force production enabling myofibroblasts to close wound sites or to induce tissue contracture during disease. Elucidation of the factors that regulate the evolution and function of myofibroblasts may thus be useful for identification of therapeutic approaches to counteract the development of pathological conditions mediated by myofibroblasts. 

Epithelial cells, if presented with appropriate cues, can transition to a myofibroblast phenotype through an epithelial-mesenchymal transition (EMT). Transforming growth factor (TGF)-β1, a potent inducer of EMT, promotes the loss of epithelial features, including apico-basal polarity and intercellular contacts, and the gain of mesenchymal properties including increased migratory capacity and contractility. Furthermore, during EMT cells exhibit dramatic morphological changes. These phenotypic changes are accompanied by changes in gene expression patterns including reduced expression of epithelial markers such as E-cadherin and cytokeratins and upregulation of mesenchymal markers including vimentin [7]. Further progression of EMT can lead to the induction of a myogenic program and the *de novo* expression of αSMA resulting in the development of myofibroblasts [8].

Adhesion to extracellular matrix (ECM) controls cell morphology and adhesion to some ECM components can regulate EMT [9–12]. Indeed, in some experimental systems cell morphological changes induce features of EMT [13,14]. Cell morphology can also be modulated by physical properties of the microenvironment including matrix rigidity [15]. Biophysical cues are implicated in the regulation of TGFβ1-induced EMT as rigid matrices promote EMT and compliant matrices block EMT in mammary, kidney, and lung epithelial cells [9,16]. A recent study demonstrated that micropatterned epithelial cells exhibit high expression levels of cytokeratins across a range of cell spread areas and that TGFβ1 treatment induces downregulation of cytokeratins and upregulation of vimentin across the same range of cell spread areas [17]. However, it is not clear whether TGFβ signaling and cell shape together regulate the induction of myogenic and cytoskeletal regulatory proteins during the development of myofibroblasts from epithelial cells.

Serum response factor (SRF) regulates the transcription of genes associated with adhesion and differentiation [18–20] and has been implicated in the control of the myofibroblast phenotype [21,22]. The transcriptional activity of SRF is regulated by a variety of cofactors, including the myocardin-related transcription factor (MRTF) family members MRTF-A and -B (also known as MAL, BSAC, and MKL1/2) [20]. The subcellular localization and activity of MRTFs are in part controlled by their association with monomeric actin (G-actin). Shifts in actin polymerization induce dissociation of MRTFs from G-actin thus allowing for MRTFs to localize to the cell nucleus to interact with SRF to promote gene expression. MRTFs play an important role in regulating a variety of cell fates and behaviors including EMT [21,23–26], experimental metastasis [27], and myofibroblast activation in response to myocardial infarction [28]. Previous studies have found that the nuclear localization of MRTF-A is sensitive to endogenous and exogenous forces [24,29–32]. Cell shape can modulate cytoskeletal tension, thus, MRTF signaling during TGFβ1-induced EMT may be promoted by changes in cellular morphology that characterize the progression of EMT.

In this study, we sought to determine whether the expression of cytoskeletal genes induced in epithelial cells by TGFβ1 depends on cell adhesion and cell shape. Epithelial cells exhibit increased cell spreading upon TGFβ1-induced EMT. Increased cellular tension is thought to be necessary for the development of myofibroblasts [33,34]; thus, we hypothesized that changes in cell spreading may be necessary for the increased expression of cytoskeletal proteins and the development of myofibroblasts from epithelial cells. We found that mammary epithelial cells that were cultured in conditions that permitted cell spreading exhibited increased expression of αSMA, caldesmon, and tropomyosin while limiting cell adhesion abrogated upregulation of the myofibroblast program. We identify MRTF-A as a key determinant in the cell shape-dependent control of cytoskeletal and myofibroblast-specific gene expression during TGFβ1-induced EMT. These results suggest that cell adhesion and shape in combination with MRTF-A signaling play an important role in the regulation of TGFβ1-induced myofibroblast transdifferentiation from epithelial cells.

## Materials and Methods

### Cell culture and reagents

NMuMG cells were obtained from American Type Culture Collection (ATCC CRL-1636) and were cultured in DMEM supplemented with 10% fetal bovine serum (Atlanta Biologicals), 10 µg/ml insulin (Sigma), and 50 µg/ml gentamicin (Life Technologies). Cells were serum starved overnight and then treated with 10 ng/ml recombinant human TGFβ1 (R&D Systems) or carrier solution for 48 h. For inhibitor studies, cells were treated with: Blebbistatin (10 µM, Sigma); Y-27632 (10 µM, Tocris); CCG-1423 (7.5 µM, Enzo). For MRTF-A localization studies, cells were treated with TGFβ1 as described followed by leptomycin B (1 ng/ml, Enzo), a nuclear export inhibitor, for 1 h prior to cell fixation.

### Fabrication of micropatterned substrata

Microfabricated substrata were created using a modified microcontact printing stamp-off procedure [35,36]. Briefly, poly(dimethylsiloxane) (PDMS; Dow Corning Corporation) stamps were cast from master silicon molds generated by photolithography. The PDMS stamps and PDMS-coated glass coverslips were UV-oxidized for 7 minutes. Featureless PDMS slabs were coated with 25 µg/ml fibronectin (BD Biosciences) for 2 h, rinsed with 1× phosphate buffered saline (PBS), dried thoroughly with nitrogen, and then brought into conformal contact with the template stamp to remove fibronectin from the PDMS slab. PDMS-coated glass coverslips were then stamped with fibronectin from the PDMS slab and blocked with 1% Pluronics F127 (Sigma) for 30 min. Coverslips were rinsed with PBS and then seeded with cells. Finally, samples were rinsed after 15 min to remove non-adherent cells. 

### Plasmids and transfections

Human FLAG-tagged MRTF-A (p3xFLAG-MRTF-A) [37], MRTF-A-ΔN, and the p3xFLAG-CMV-7.1 vector with a subcloned YFP were obtained from Celeste Nelson (Princeton University) [24]. MRTF-A-ΔC was generated using a QuikChange II XL Site-Directed Mutagenesis Kit (Agilent Technologies) to remove 903 bp from the carboxyl terminus of MRTF-A [37]. Cells were transfected with plasmids using Lipofectamine LTX with Plus Reagent (Life Technologies) following the manufacturer’s protocol.

### Immunofluorescence staining

For staining of αSMA, samples were fixed with 1:1 methanol/acetone at -20 °C for 10 min. For all other stains, samples were rinsed with PBS and then fixed with 4% paraformaldehyde at room temperature for 15 min. Following fixation, samples were rinsed with PBS, permeabilized with 0.1% Triton X-100, blocked with 10% goat serum (Sigma), and incubated with the following primary antibodies: αSMA (1A4, Sigma), tropomyosin (TM311, Sigma), caldesmon (E89, Abcam), pSmad3 (D12E11, Cell Signaling), and MRTF-A (H140, Santa Cruz Biotechnology). Samples were then rinsed with PBS and incubated with Alexa Fluor-conjugated secondary antibodies (Life Technologies). Nuclei were counterstained with Hoechst 33342 (Life Technologies). pSmad3 staining was performed following a 1 h treatment with TGFβ1. For staining of filamentous actin, samples were fixed with 4% paraformaldehyde and then incubated with Alexa Fluor 594 phalloidin (Life Technologies). All samples were mounted on glass coverslides using Fluormount-G (Electron Microscopy Sciences). 

### Western blotting

NMuMG cells were lysed in ice cold RIPA buffer with protease and phosphatase inhibitors (Pierce). Equal amounts of lysate were separated on a 10% tris-glycine gel (Life Technologies) and transferred to a PVDF membrane. Membranes were blocked with 5% non-fat dry milk and analysis was performed with primary antibodies against αSMA (1:2500; Sigma), caldesmon (1:10000; Abcam), tropomyosin (1:700; Sigma), vimentin (1:500; Sigma), E-cadherin (1:1000; Cell Signaling), α-tubulin (1:1000, Sigma), and βActin (1:1000; Cell Signaling). HRP-conjugated secondary antibodies (1:2000; Cell Signaling) and SuperSignal West Pico Chemiluminescent Substrate (Pierce) were used for detection. Blots were imaged using a FluorChem FC2 system (Cell Biosciences). Densitometric analysis was performed using ImageJ software.

### Quantitative real-time PCR

Total RNA was isolated from cells using a RNeasy Plus kit (Qiagen) followed by cDNA synthesis using a High Capacity cDNA Reverse Transcription Kit (Applied Biosystems). Transcript levels were measured using Taqman assays (Life Technologies) on an Applied Biosystems 7300 Real-time PCR System. For each sample, melt curve analysis was performed to ensure a single PCR product and mRNA expression was normalized to the expression of cyclophilin.

### Microscopy and analysis

Samples were imaged using a 40× air objective on a Nikon Eclipse Ti-E inverted fluorescence microscope equipped with a Photometrics CoolSNAP HQ^2^ CCD camera or with an Olympus FV1000 confocal microscope. Cell area, aspect ratio, and integrated fluorescence intensity were measured using ImageJ software. For scoring of protein expression, at least 50 cells were analyzed per condition. Relative levels were computed with respect to 400 μm^2^ control samples for varying spread area or with respect to shape factor 1 control samples for varying cell aspect ratio. MRTF-A subcellular localization was determined by comparing the nuclear and cytoplasmic fluorescence intensities within cells. Cells showing a mean nuclear intensity that was two-fold greater than the mean cytoplasmic intensity were classified as exhibiting nuclear localized MRTF-A.

Samples were compared by either a student t-test or by ANOVA followed by the Bonferroni correction for multiple comparisons using Kaleidagraph 4.1 software. Differences were considered significant for p < 0.05. A minimum of three independent experiments were performed for all studies. All reported values are mean ± standard error of the mean.

## Results

### Cell spreading is necessary for TGFβ1-induced expression of myofibroblast markers

**Figure 1:**
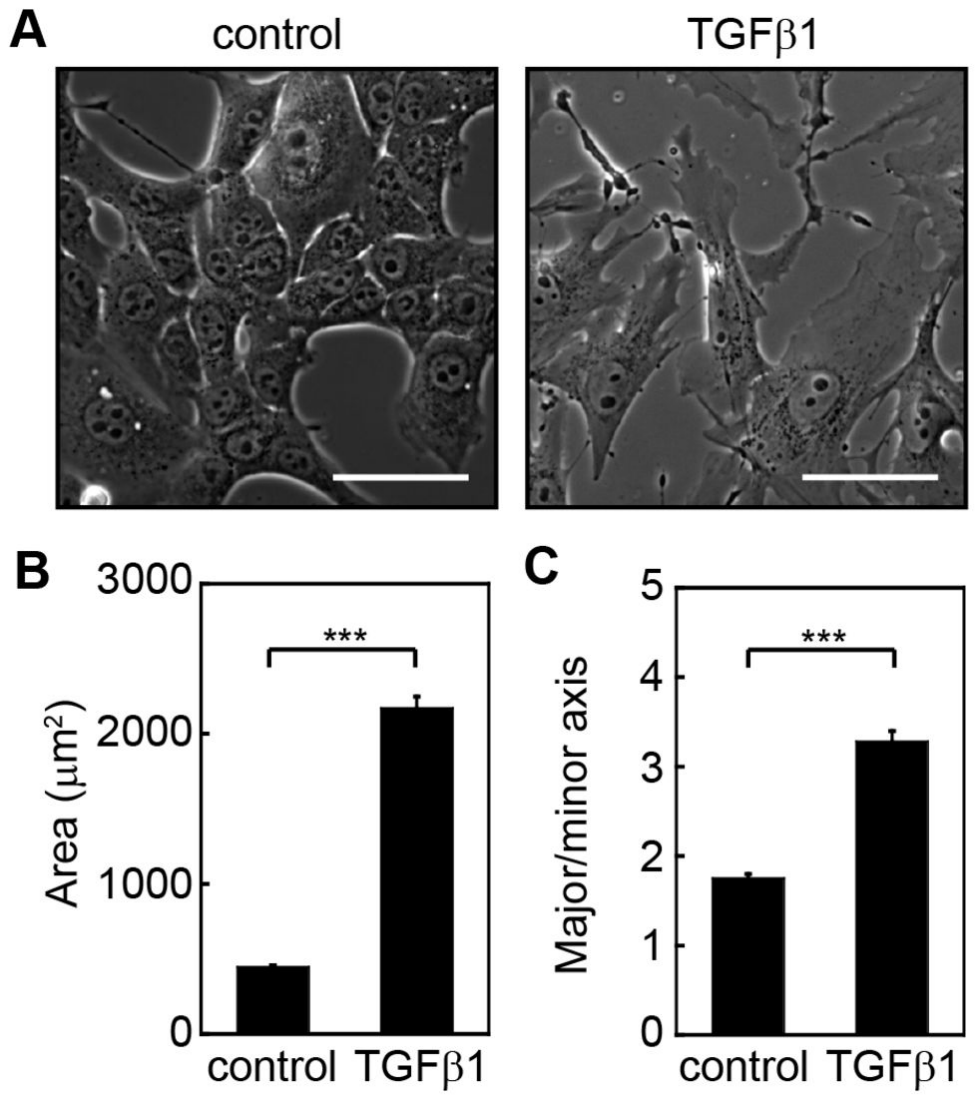
TGFβ1-induced EMT correlates with increased cell spreading and elongation. (A) Phase contrast images of NMuMG cells treated with and without TGFβ1. Quantification of (B) projected cell area and (C) cell elongation for NMuMG cells treated with and without TGFβ1. ***p < 0.001. n > 150 cells. Scale bars, 50 μm.

**Figure 2:**
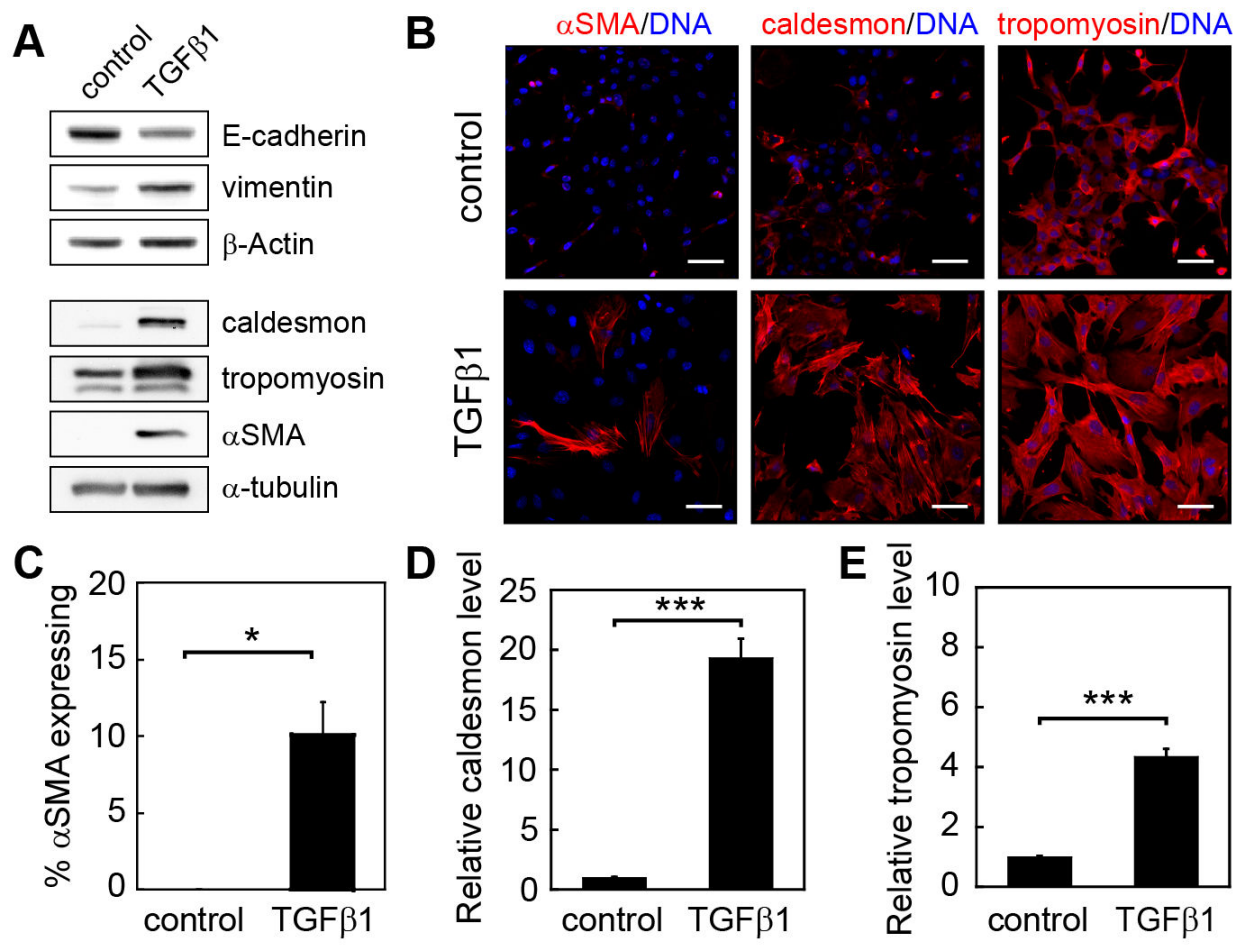
TGFβ1 induces the expression of myofibroblast markers and cytoskeletal associated proteins in NMuMG cells. (A) Western blot for EMT markers and cytoskeletal associated proteins in NMuMG cells after 48 h treatment with or without TGFβ1. (B) Immunofluorescence staining for αSMA, caldesmon, and tropomyosin in control and TGFβ1-treated samples. Quantification of the percentage of cells expressing (C) αSMA and the relative levels of (D) caldesmon and (E) tropomyosin expression in control and TGFβ1-treated samples. Relative levels are computed in comparison to control samples. *p < 0.05, ***p < 0.001. Scale bars, 50 μm.

Induction of EMT by TGFβ1 results in significant changes in cell morphology with epithelial cells transitioning from a cuboidal, cobblestone morphology to an elongated, fibroblast-like shape (Figure 1A). Quantification of these cell shape changes reveal increased cell spreading on the underlying substratum and increased cell aspect ratio (Figure 1B,C). These morphological changes are accompanied by reduced expression of the epithelial marker E-cadherin and increased expression of the mesenchymal marker vimentin (Figure 2A and Figure S1). Likewise, TGFβ1 induces increased expression of myofibroblast markers and cytoskeletal associated proteins including αSMA, caldesmon, and tropomyosin (Figure 2). These proteins mediate assembly and stability of filamentous actin and cellular contractility [5,38,39].

**Figure 3:**
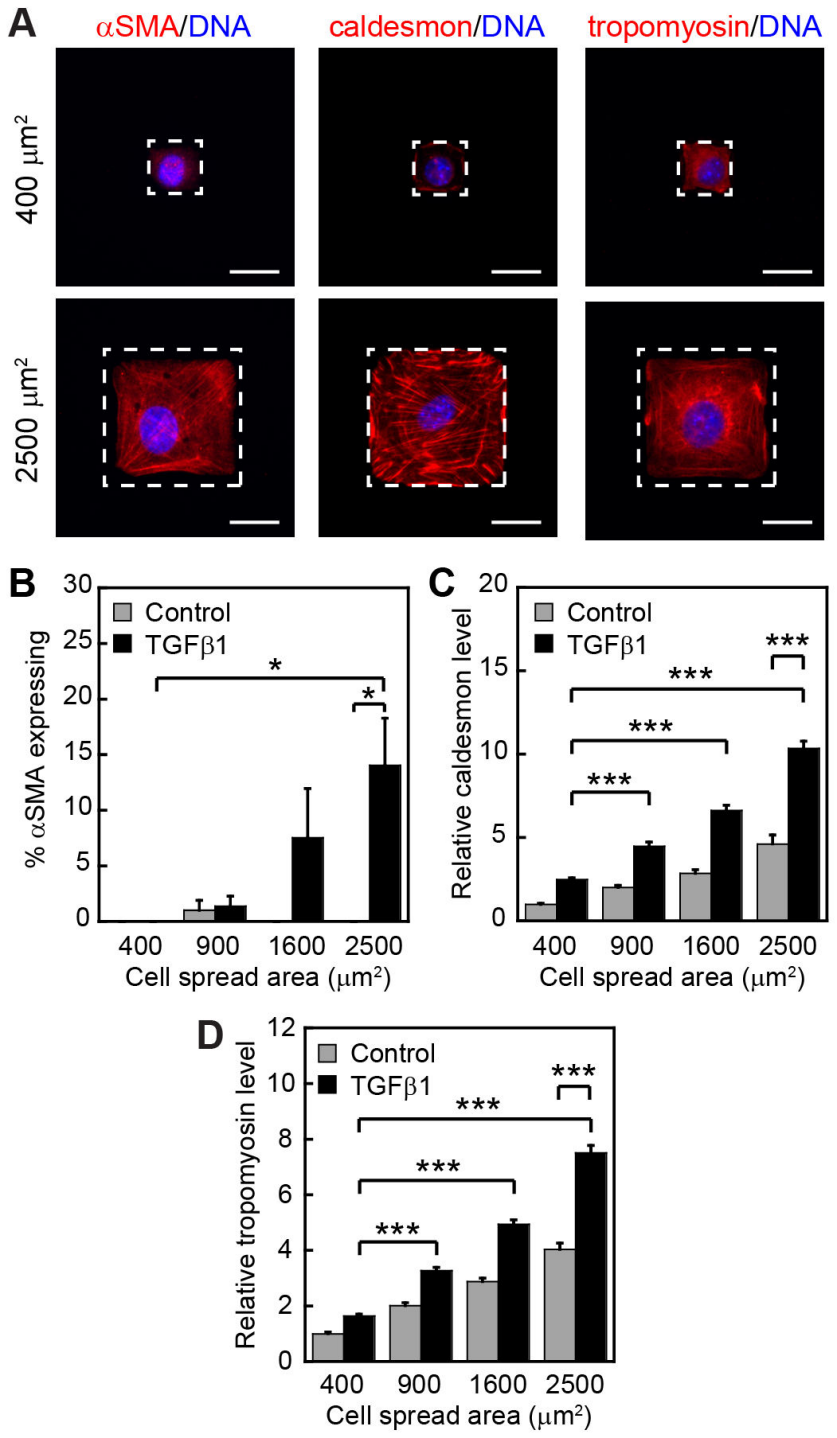
Cell spreading promotes TGFβ1-induced expression of actin binding proteins and myofibroblast markers. (A) Immunofluorescence staining of αSMA, caldesmon, and tropomyosin for TGFβ1-treated cells cultured on 400 μm^2^ and 2500 μm^2^ squares. Stamped protein islands are outlined by a white dotted line. (B) Frequency of squares with αSMA expression after treatment with and without TGFβ1. Relative levels of (C) caldesmon and (D) tropomyosin with and without TGFβ1. Relative levels are computed in comparison to 400 μm^2^ control samples. *p < 0.05; ***p < 0.001. Scale bars, 20 μm.

To determine whether TGFβ1-induced increases in cell spread area and cell aspect ratio are required for the upregulation of cytoskeletal associated proteins we employed a microcontact printing approach in order to precisely control the size and shape of individual cells. NMuMG cells were seeded onto square islands of fibronectin of varying sizes ranging from 400 μm^2^ to 2500 μm^2^ in area. These sizes encompass the range of cell spread areas exhibited by NMuMG cells on fibronectin before and after induction of EMT by TGFβ1 (Figure 1). Cells were cultured on the microfabricated substrata for 48 h in the presence or absence of TGFβ1. Cells cultured on 2500 μm^2^ islands, which permitted cell spreading, exhibited increased expression of αSMA in the presence of TGFβ1 in comparison to control cells. In contrast, cells cultured on 400 μm^2^ islands were unable to spread appreciably and did not increase expression of αSMA (Figure 3). The expression of caldesmon and tropomyosin increased moderately as a function of cell spread area in control cells, and this effect was enhanced by treatment with TGFβ1 (Figure 3C,D). These data indicate that TGFβ1 induces the expression of myofibroblast markers and cytoskeletal associated proteins preferentially in spread cells, suggesting that increased adhesion and cell spreading are key determinants in the transdifferentiation of myofibroblasts from epithelial cells.

**Figure 4:**
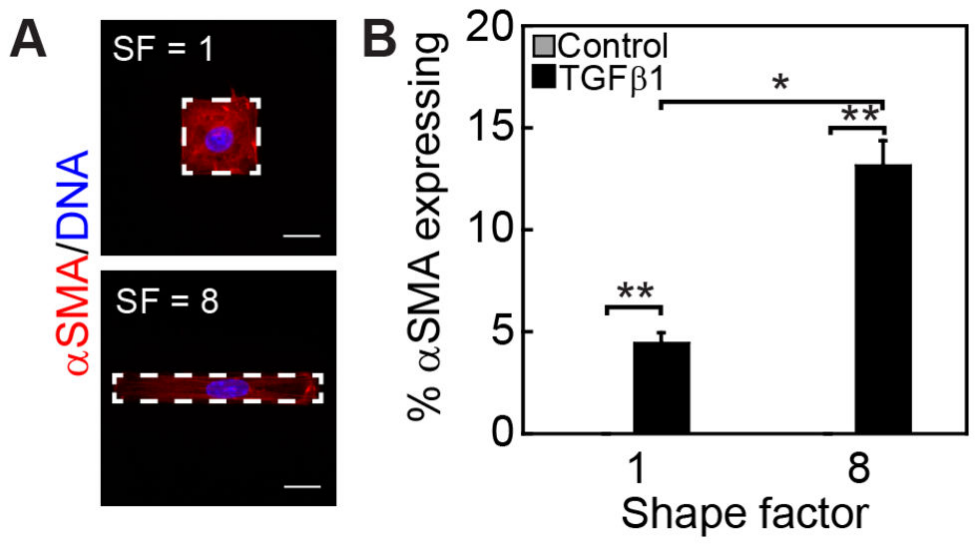
Cell shape regulates TGFβ1-induced expression of αSMA. (A) Immunofluorescence staining of αSMA for TGFβ1-treated NMuMG cells on 1600 μm^2^ islands with shape factors 1 and 8. (B) Quantification of the percentage of NMuMG cells expressing αSMA on islands with shape factors 1 and 8 after a 48 h treatment with or without TGFβ1. *p < 0.05, **p < 0.01. Scale bars, 20 μm.

Since changes in cell shape regulate cellular differentiation in other contexts [32,40], we sought to decouple this factor from cell spread area to directly probe the role of cell shape in the regulation of TGFβ1-induced cytoskeletal protein expression. To this end, we generated substrata with microcontact printed protein islands with a constant area of 1600 μm^2^ and varying shape factor. Here, shape factor is defined as the ratio of the major axis to the minor axis of the printed protein island. An increase in shape factor from a square to a rectangle mimics the cell shape changes exhibited by epithelial cells during EMT as they transition to an elongated, fibroblastoid morphology following treatment with TGFβ1 (Figure 1A, C). The percentage of cells expressing αSMA was significantly higher for cells cultured on microfabricated substrata with a shape factor of 8 compared to a shape factor of 1 (Figure 4). Similarly, the expression level of caldesmon and tropomyosin increased with increasing shape factor (Figure S2). These results demonstrate a role for cell shape in the regulation of TGFβ1-induced expression of myogenic markers and actin binding proteins in epithelial cells.

In the TGFβ1 canonical signaling pathway, TGFβ ligands bind to receptors displayed on the cell surface which stimulate phosphorylation and subsequent nuclear translocation of Smad proteins [41]. The observed increase in the levels of myofibroblast markers as a function of cell shape could thus be a result of differences in the activation of the TGFβ1 canonical signaling pathway. Immunoflourescence analysis revealed that pSmad3 localized to the cell nucleus in all cells treated with TGFβ1 regardless of cell spread area (Figure S3), suggesting that activation of Smad signaling is not disrupted by restricting cell spreading. Therefore, the observed increase in the expression of myofibroblast markers as a function of cell shape is likely regulated by other signaling mechanisms.

### Expression of myofibroblast markers requires increases in intracellular tension

**Figure 5:**
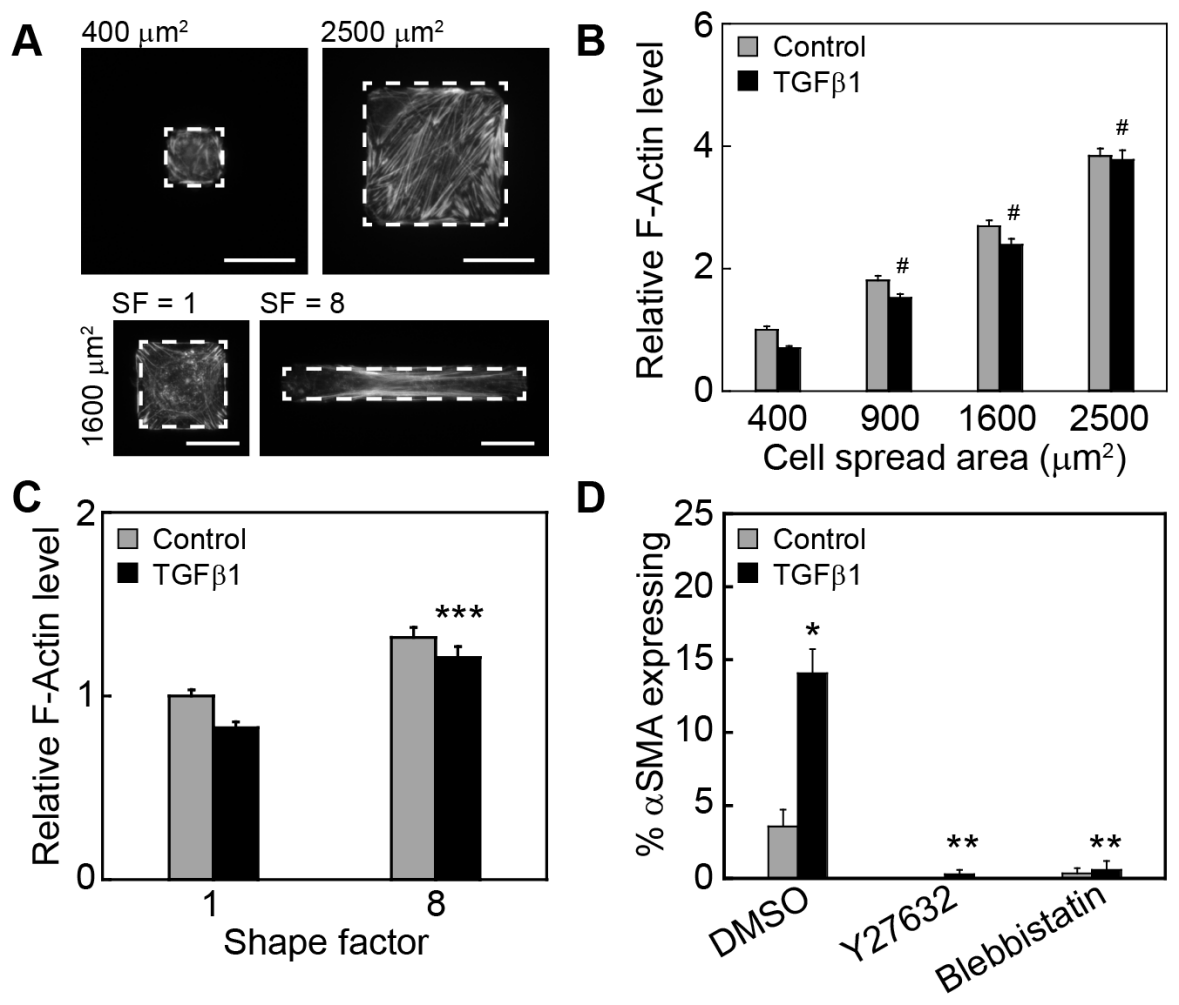
Cell shape regulates the organization of the actin cytoskeleton in NMuMG cells. (A) Fluorescence staining for F-actin in TGFβ1-treated NMuMG cells cultured on 400 μm^2^ and 2500 μm^2^ squares and on 1600 μm^2^ islands with shape factor 1 and 8. Quantification of the relative total integrated intensity of phalloidin in NMuMG cells of (B) varying area (^#^p < 0.001 in comparison to 400 μm^2^, TGFβ1 treatment) and (C) shape factor (***p < 0.001 in comparison to SF1, TGFβ1 treatment). (D) Percentage of cells cultured on 2500 μm^2^ squares that express αSMA after simultaneous treatment with TGFβ1 and contractility inhibitors (*p < 0.05 in comparison to DMSO control; **p < 0.01 in comparison to DMSO TGFβ1). Scale bars, 25 μm.

During TGFβ1-induced EMT cells exhibit dramatic cytoskeletal reorganization that is mediated by signaling through the Rho GTPase pathway [42]. To elucidate whether the organization of the actin cytoskeleton contributes to cell shape control of TGFβ1-induced EMT the actin cytoskeleton was visualized using fluorescently labeled phalloidin, a molecule which selectively binds to actin filaments [43]. Control and TGFβ1 treated cells cultured on 2500 μm^2^ islands exhibited actin filaments (F-actin) while restricting cell adhesion by culturing cells on smaller islands blocked substantial actin filament assembly (Figure 5A). Quantification of phalloidin fluorescence revealed that levels of F-actin increased with an increase in cell adhesion and spreading for both control and TGFβ1 treated cells (Figure 5B). These results are consistent with previous studies which found that cells with increased cell spreading contain increased filamentous actin and higher levels of cytoskeletal tension [44,45]. An increase in F-actin levels was not observed for cells upon treatment with TGFβ1 in comparison to control cells of the same spread area at 48 h following treatment with TGFβ1. Similarly, levels of F-actin increased for cells cultured on microfabricated substrata with a shape factor of 8 compared to a shape factor of 1 for TGFβ1 treated and control cells (Figure 5C). Decreasing contractile tension by treatment with the non-muscle myosin ATPase inhibitor blebbistatin or the ROCK inhibitor Y27632 reduced the percentage of cells expressing αSMA on 2500 μm^2^ islands in the presence of TGFβ1 (Figure 5D). These data support previous findings demonstrating that signaling through RhoA/ROCK mediates TGFβ1-induced actin cytoskeletal rearrangements and is necessary for induction of EMT [42]. Moreover, our results suggest that the differential organization of the actin cytoskeleton and increases in cellular tension may play an important role in mediating control of the expression of myofibroblast markers by cell shape.

### Cell shape controls the expression of myofibroblast markers by governing the subcellular localization of MRTF-A

**Figure 6:**
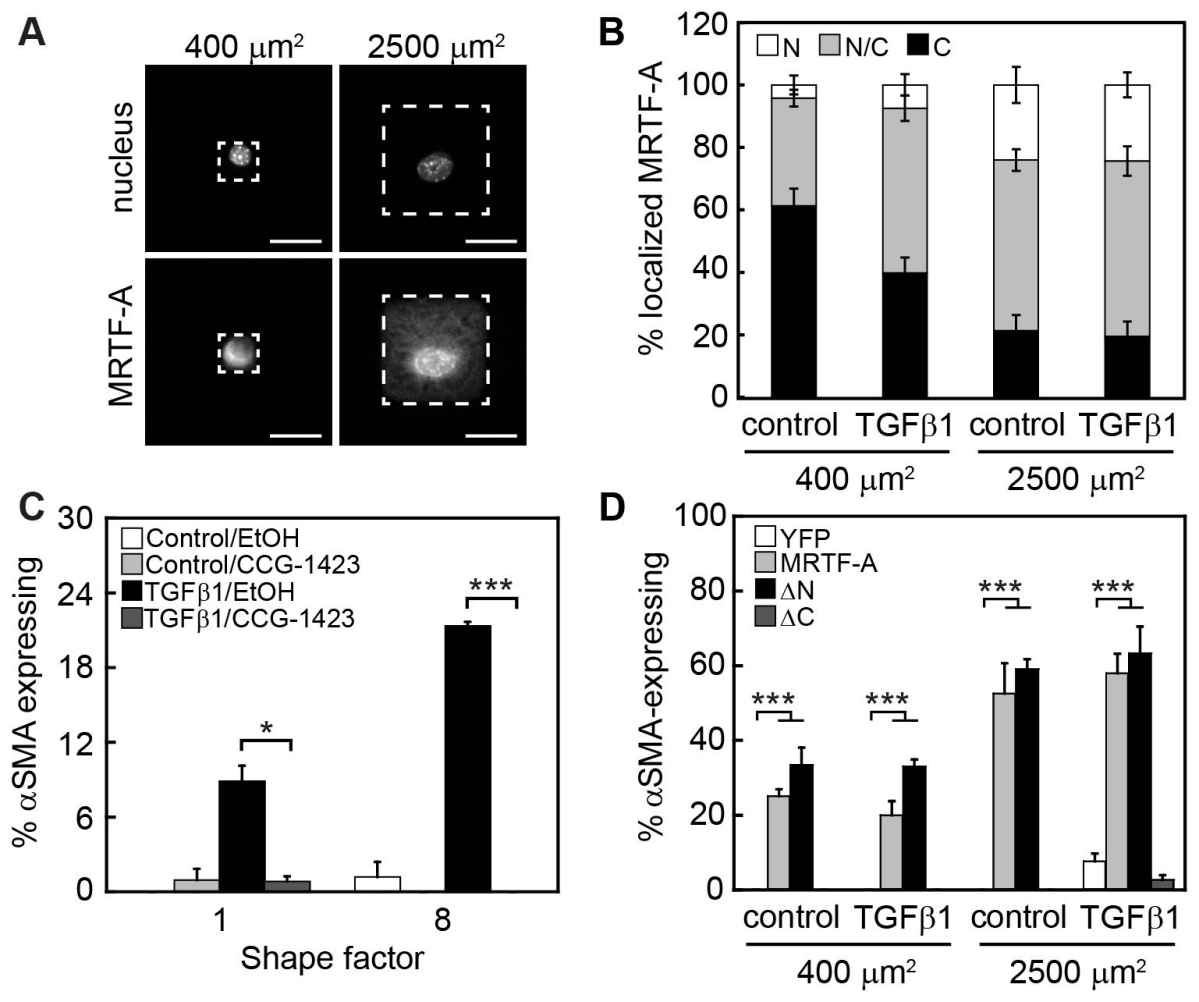
Myocardin-related transcription factor signaling controls cell shape-dependent induction of αSMA by TGFβ1. (A) Immunofluorescence staining for nuclei and MRTF-A in TGFβ1-treated NMuMG cells cultured on 400 μm^2^ and 2500 μm^2^ squares. (B) Quantification of the percentage of cells with nuclear (N), pan-cellular (N/C), and cytoplasmic (C) MRTF-A as a function of cell spread area. (C) Quantification of the percentage of cells expressing αSMA after simultaneous treatment with TGFβ1 and ethanol vehicle or CCG-1423. *p < 0.01; ***p < 0.001 compared to ethanol control. Overexpression of Flag-tagged MRTF-A and Flag-tagged MRTF-A-ΔN increase the expression of αSMA. (D) Quantification of the percentage of cells expressing αSMA for YFP, MRTF-A, MRTF-A-ΔC, and MRTF-A-ΔN transfected NMuMG cells treated with TGFβ1 or control vehicle. ***p < 0.001 compared to YFP. Scale bars: 25 μm.

Recent studies have linked cell adhesion and actin dynamics to the serum response factor (SRF) signaling pathway [32,46–48]. A subset of SRF target genes is regulated by Rho-actin signaling and by shifts in the polymerization of actin [18]. Myocardin related transcription factors (MRTF)-A and –B, cofactors of SRF, mediate this effect [46]. Notably, MRTF-A regulates the transcription of αSMA [49], a key marker of the myofibroblast phenotype. Thus, we hypothesized that MRTF-A signaling might be involved in cell shape-dependent transdifferentiation of myofibroblasts from epithelial cells. MRTF-A was observed to localize to the nucleus in control and TGFβ1-treated cells that were permitted to spread, however, restricting cell spreading reduced the percentage of cells that exhibited MRTF-A nuclear localization (Figure 6A,B). The increase in MRTF-A nuclear localization in cells cultured on large islands in comparison to small islands correlates with observed increases in F-actin and in expression of αSMA, caldesmon, and tropomyosin in TGFβ1-treated cells. Treatment with CCG-1423, an inhibitor that blocks the interaction between SRF and MRTF-A [50], decreased the percentage of TGFβ1 treated cells that expressed αSMA (Figure 6C). Likewise, CCG-1423 blocked the increased expression of caldesmon and tropomyosin in TGFβ1 treated cells (Figure S4). These data suggest that cell adhesion and shape control TGFβ1-induced expression of myofibroblast markers by cooperating with SRF/MRTF-A signaling and by regulating the nuclear localization of MRTF-A.

Overexpression of MRTF-A and a constitutively active form of MRTF-A (MRTF-A-ΔN), which lacks the RPEL motifs that mediate binding to actin monomers [22,46], resulted in increased expression of αSMA in control and TGFβ1 treated cells cultured on both small and large islands (Figure 6D). Expression of a dominant negative form of MRTF-A (MRTF-A-ΔC), which lacks the transcriptional activation domain [37], did not promote the expression of αSMA (Figure 6D). Similar results were observed for the expression of caldesmon and tropomyosin (Figure S4). These data further support a role for MRTF-A signaling in the regulation of TGFβ1-induced expression of cytoskeletal proteins by cell adhesion and cell shape.

## Discussion

**Figure 7:**
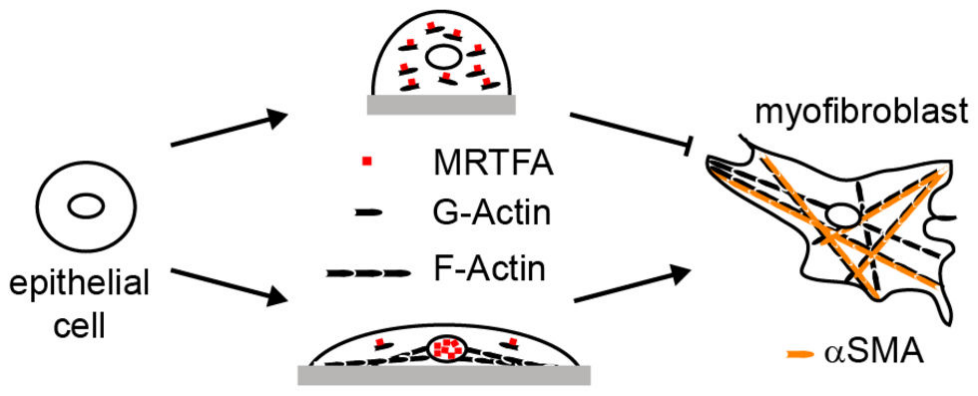
Model proposing how cell shape, MRTF-A, and TGFβ1 signaling regulate the development of myofibroblasts from epithelial cells.

In this study, we found that cell morphology regulates TGFβ1-induced expression of cytoskeletal genes during the development of myofibroblasts from epithelial cells. Culture of epithelial cells in shapes that promoted increased actin filament assembly and cellular contractility led to the induction of a myogenic program by TGFβ1. In contrast, cell shapes that prevented actin polymerization blocked TGFβ1-mediated upregulation of myofibroblast markers. Our findings suggest a model whereby cell adhesion and shape modulate the relative levels of monomeric and filamentous actin within the cell thereby controlling the nuclear localization of MRTF-A, as shown in Figure 7. Once in the nucleus, MRTF-A cooperates with other TGFβ1-induced signaling cascades to regulate the expression of cytoskeletal associated proteins.

Changes in cell morphology have previously been reported to control a variety of cell behaviors including cell division [51], proliferation and apoptosis [52], migration [53], and differentiation [32,40,54,55]. Our results show that culture of epithelial cells on small islands blocks TGFβ1-induced increases in the expression of αSMA while culture on large islands that permit cell spreading promotes the myofibroblast phenotype. Decreasing cytoskeletal tension in cells that were permitted to spread blocked the upregulation of αSMA by TGFβ1. These results are consistent with previous studies that have demonstrated a role for RhoA/ROCK signaling and cytoskeletal tension in the induction of αSMA expression and EMT and in the control of myofibroblast properties [34,42]. Given the roles of cell shape and Rho/ROCK signaling in regulating a variety of other cell behaviors including cell cycle progression [56] and the differentiation of mesenchymal stem cells [40,54], it will be interesting to determine whether SRF/MRTF-A signaling acts in concert with cell shape to control these cell processes. 

In addition to changes in cell spread area, cell elongation is also known to modulate cell fate and function including stem cell differentiation[32], macrophage phenotype[57], and contractile force[58]. We find that increased cell elongation for a fixed cell spread area promotes increased expression of αSMA and cytoskeletal associated proteins in TGFβ1 treated cells. Previous studies have demonstrated that cell elongation induces stress fiber alignment in endothelial cells[59] and correlates with increased focal adhesion size and increased traction forces in fibroblasts[60]. Activation of focal adhesion kinase (FAK), a component of focal adhesions, is required for TGFβ1-induced EMT[61]. Furthermore, FAK cooperates with gelsolin to mediate force-induced nuclear localization of MRTF-A and αSMA expression in fibroblasts[29]. Thus, it is possible that cell elongation promotes increased expression levels of αSMA in TGFβ1 treated epithelial cells through increased nuclear localization of MRTF-A or increased focal adhesion signaling.

Loss of cell-cell contacts is an early event that occurs during EMT. Previous studies have proposed a two-hit model whereby disruption of intercellular contacts in combination with TGFβ enhances MRTF-A signaling and myofibroblast marker expression by promoting prolonged nuclear localization of MRTF-A [8,62]. Our results suggest that a lack of cell-cell contact is not sufficient to induce a myogenic program in epithelial cells in the presence of TGFβ1. We find that in addition to these factors, cell adhesion and cell shape control the development of myofibroblasts from epithelial cells.

Recent studies have demonstrated a link between cell adhesion and SRF signaling, with increased cell spreading enhancing SRF promoter activity [48,63]. Our findings are consistent with these results, as we show that MRTF-A nuclear localization and the expression levels of SRF/MRTF-A target genes are enhanced by cell spreading. However, our observation that significant increases in the expression of αSMA are not observed in control cells that are permitted to spread (even though MRTF-A localizes to the nucleus in a fraction of these cells) suggests that other factors are involved in regulating the transcriptional activity of MRTFs in the context of TGFβ1-induced EMT. Our data suggest that translocation of MRTF-A to the cell nucleus in spread epithelial cells is necessary but not sufficient to induce the expression of all SRF/MRTF target genes. In light of this, we posit that increases in cell adhesion and spreading predispose epithelial cells to enhanced MRTF transcriptional activity by promoting increases in cytosolic actin polymerization which then drives MRTF to the cell nucleus. Once in the nucleus, the activity of MRTF may be determined by a variety of factors including the relative levels of negative regulators such as intranuclear G-actin [64] or Smad3 [8]. Indeed, activation of nuclear mDia can induce polymerization of nuclear actin and SRF activity [65]. Recent studies have also shown that the level of Smad3, a protein which binds to MRTFs [26] and subsequently inhibits the transcriptional activity of MRTFs [8], is reduced in epithelial cells following treatment with TGFβ1 [8,62]. Further studies are necessary to identify the key signaling factors involved in regulating the cell shape-dependent transcriptional activity of MRTF-A during TGFβ1-induced EMT.

Although our results suggest an important role for MRTF-A signaling in the regulation of TGFβ1-induced gene expression by cell shape, we cannot entirely dismiss the possibility that other factors may also contribute to cell shape effects on TGFβ1 mediated development of myofibroblasts from epithelial cells. For example, it is possible that cell shape may impact the transcription and translation of EMT associated genes. Cell shape regulates nuclear morphology[59,63,66] and cell rounding leads to histone deacetylation in mammary epithelial cells[67] while cell elongation promotes histone acetylation in mesenchymal stem cells[68]. Additionally, the subcellular localization of mRNAs can regulate gene expression[69,70]. Interestingly, mRNAs have been observed to associate with actin filaments and it has been suggested that this may facilitate translation[71–73]. Thus, it is possible that differences in the cytoskeletal architecture within rounded and spread cells may lead to differences in mRNA transport and translation. A recent study demonstrated that mammary epithelial cells express high levels of cytokeratins when cultured on both small and large ECM islands and TGFβ1 treatment induces downregulation of cytokeratins and upregulation of vimentin across a range of cell spread areas[17]. Therefore, although cell shape may affect the transcriptional and translational activity of some genes, these results suggest that restricting cell spreading does not globally downregulate the expression of all proteins associated with EMT. 

Overall, our findings demonstrate that the combined effects of cell shape and TGFβ1 signaling are critical for MRTF activity and increased expression of myofibroblast markers during EMT. In vivo, EMT occurs during normal morphogenic processes of the embryo and contributes to pathological conditions including fibrosis and cancer. Whether cell shape and MRTF signaling regulate aspects of EMT in all of these biological settings is yet to be determined. Analysis of the precise interplay between cell adhesion and MRTF signaling will permit a clearer view of how biochemical cues and mechanical forces act in concert to influence gene expression.

## Supporting Information

Figure S1 **TGFβ1 induces downregulation of epithelial markers and upregulation of mesenchymal markers in NMuMG cells**. (A) Densitometric analysis of western blots from Figure 2. (B) Transcript levels for EMT markers and cytoskeletal associated proteins determined by quantitative real-time PCR. *p < 0.05, **p < 0.01, ***p < 0.001.
(TIF)

Figure S2 **Cell shape regulates TGFβ1-induced expression of caldesmon and tropomyosin.** (A) Immunofluorescence staining for caldesmon and tropomyosin for TGFβ1-treated NMuMG cells on islands with shape factors 1 and 8. Relative levels of (B) caldesmon and (C) tropomyosin for cells cultured with and without TGFβ1 in comparison to shape factor 1 control. *p < 0.001. Scale bars, 20 μm.
(TIF)

Figure S3 **TGFβ1-induced Smad signaling in NMuMG cells as a function of cell spread area**. Immunostaining for pSmad3 and nuclei for NMuMG cells treated with TGFβ1 or control vehicle. Scale bar, 25 μm.
(TIF)

Figure S4 **Myocardin-related transcription factor signaling controls cell shape-dependent expression of caldesmon and tropomyosin by TGFβ1.** Quantification of the relative levels of (A) caldesmon and (B) tropomyosin after simultaneous treatment with TGFβ1 and ethanol vehicle or CCG-1423. Relative levels are computed in comparison to shape factor 1 control. *p < 0.05, ***p < 0.001. Overexpression of Flag-tagged MRTF-A and Flag-tagged MRTF-A-ΔN increase the expression of caldesmon and tropomyosin. Quantification of the relative levels of (C) caldesmon and (D) tropomyosin for YFP, MRTF-A, MRTF-A-ΔC, and MRTF-A-ΔN transfected NMuMG cells treated with TGFβ1 or control vehicle. Relative levels are computed in comparison to 900 μm^2^ control. ***p < 0.001 compared to YFP.
(TIF)
