# Supplementary figures and images for: Cell Adhesion and Shape Regulate TGF-Beta1-Induced Epithelial-Myofibroblast Transition via MRTF-A Signaling
Source: PLoS One. 2013 Dec 10;8(12):e83188. doi: 10.1371/journal.pone.0083188 (PMC3858353; doi:10.1371/journal.pone.0083188)

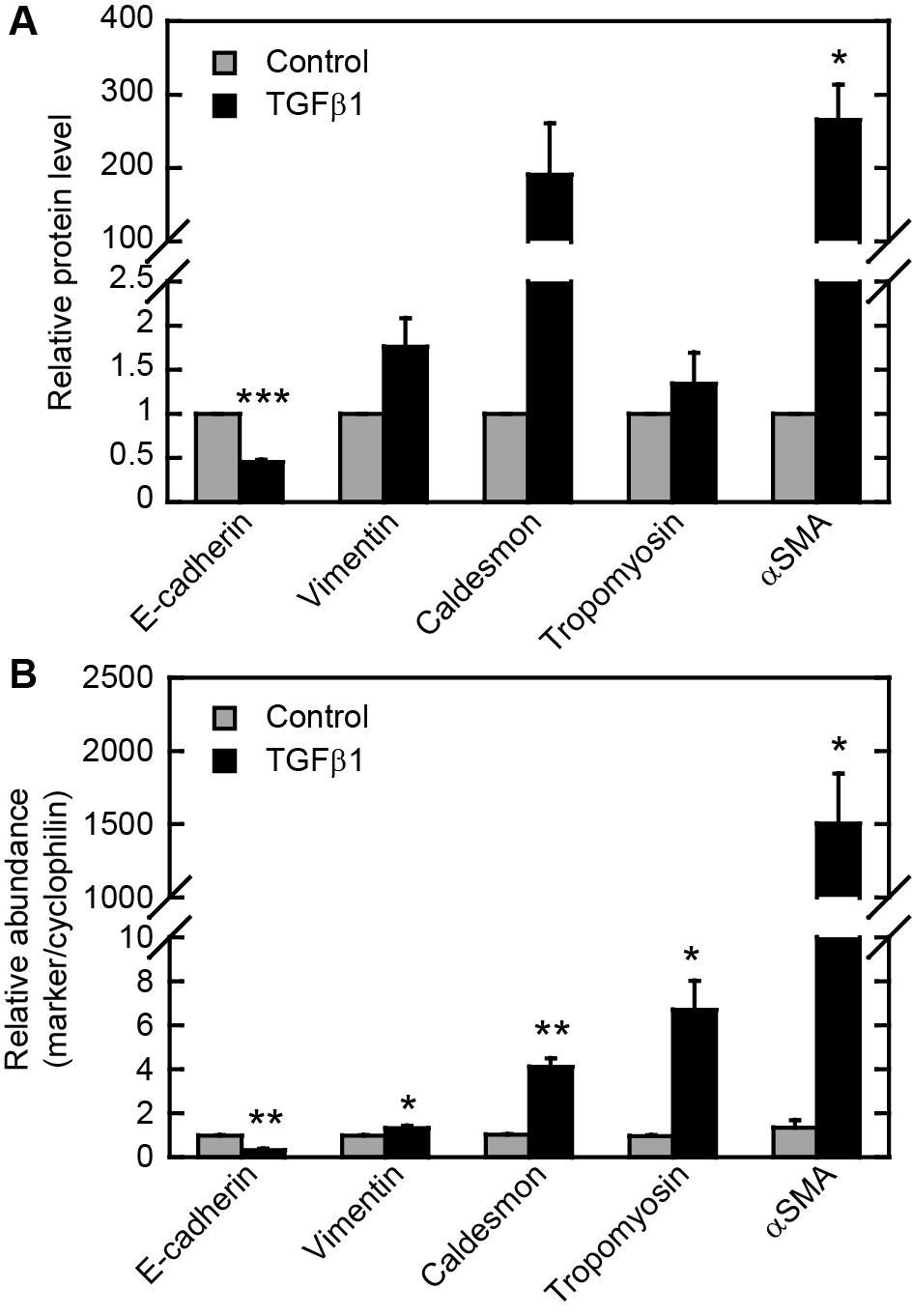

Supplement: Figure S1 — TGFβ1 induces downregulation of epithelial markers and upregulation of mesenchymal markers in NMuMG cells. (A) Densitometric analysis of western blots from Figure 2. (B) Transcript levels for EMT markers and cytoskeletal associated proteins determined by quantitative real-time PCR. *p < 0.05, **p < 0.01, ***p < 0.001. (TIF) [file pone.0083188.s001.tif]

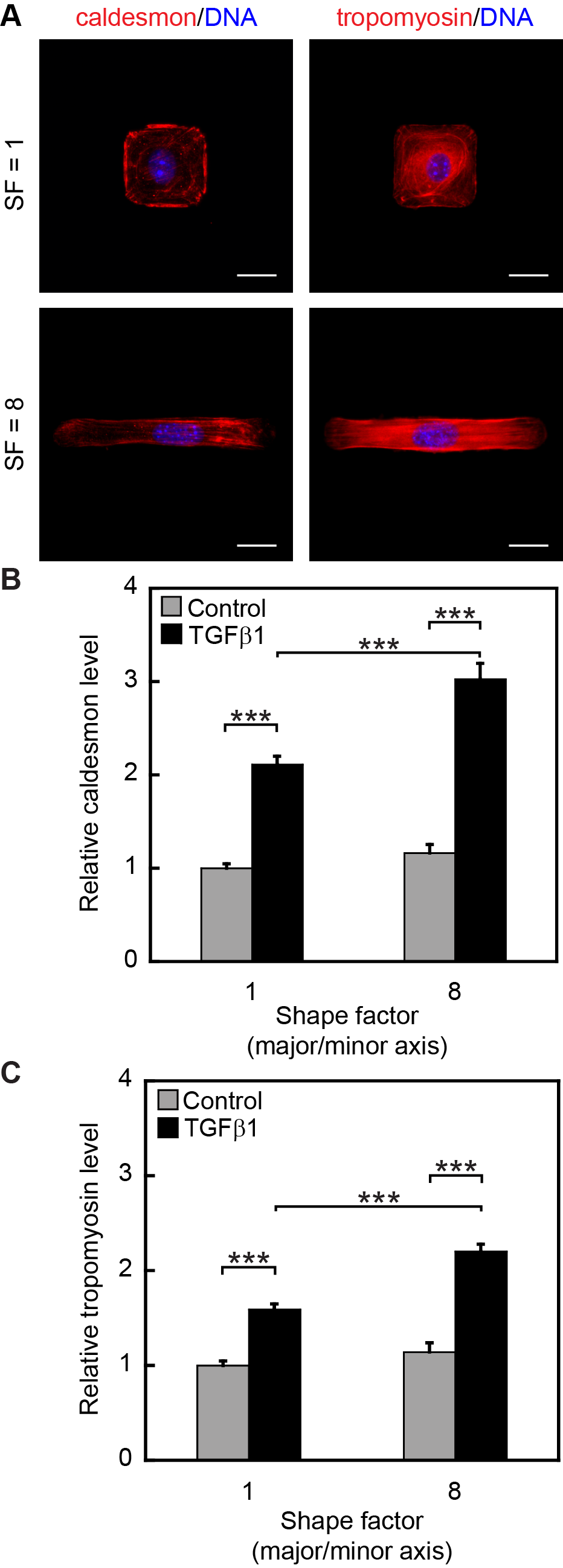

Supplement: Figure S2 — Cell shape regulates TGFβ1-induced expression of caldesmon and tropomyosin. (A) Immunofluorescence staining for caldesmon and tropomyosin for TGFβ1-treated NMuMG cells on islands with shape factors 1 and 8. Relative levels of (B) caldesmon and (C) tropomyosin for cells cultured with and without TGFβ1 in comparison to shape factor 1 control. *p < 0.001. Scale bars, 20 μm. (TIF) [file pone.0083188.s002.tif]

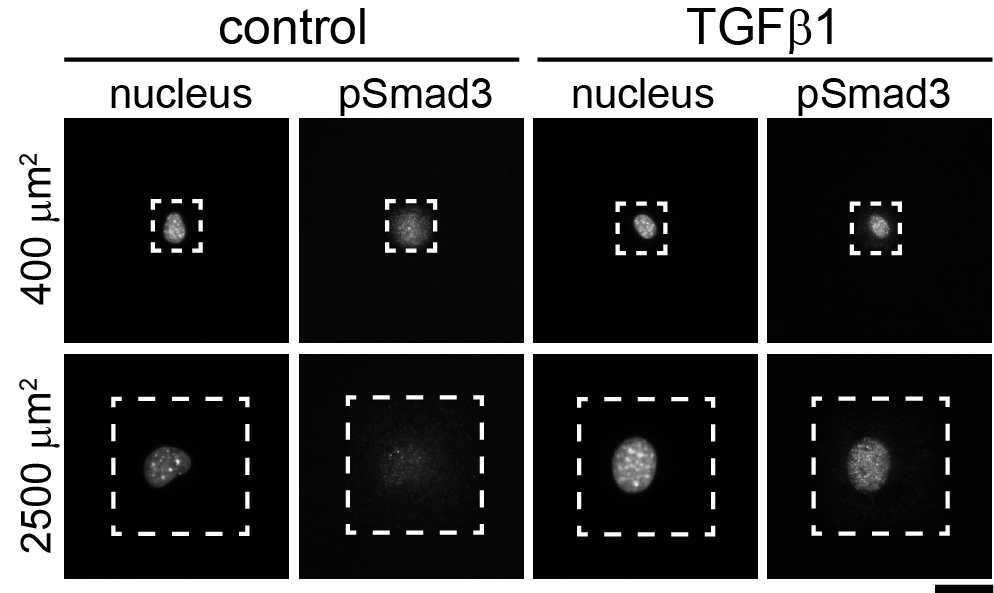

Supplement: Figure S3 — TGFβ1-induced Smad signaling in NMuMG cells as a function of cell spread area. Immunostaining for pSmad3 and nuclei for NMuMG cells treated with TGFβ1 or control vehicle. Scale bar, 25 μm. (TIF) [file pone.0083188.s003.tif]

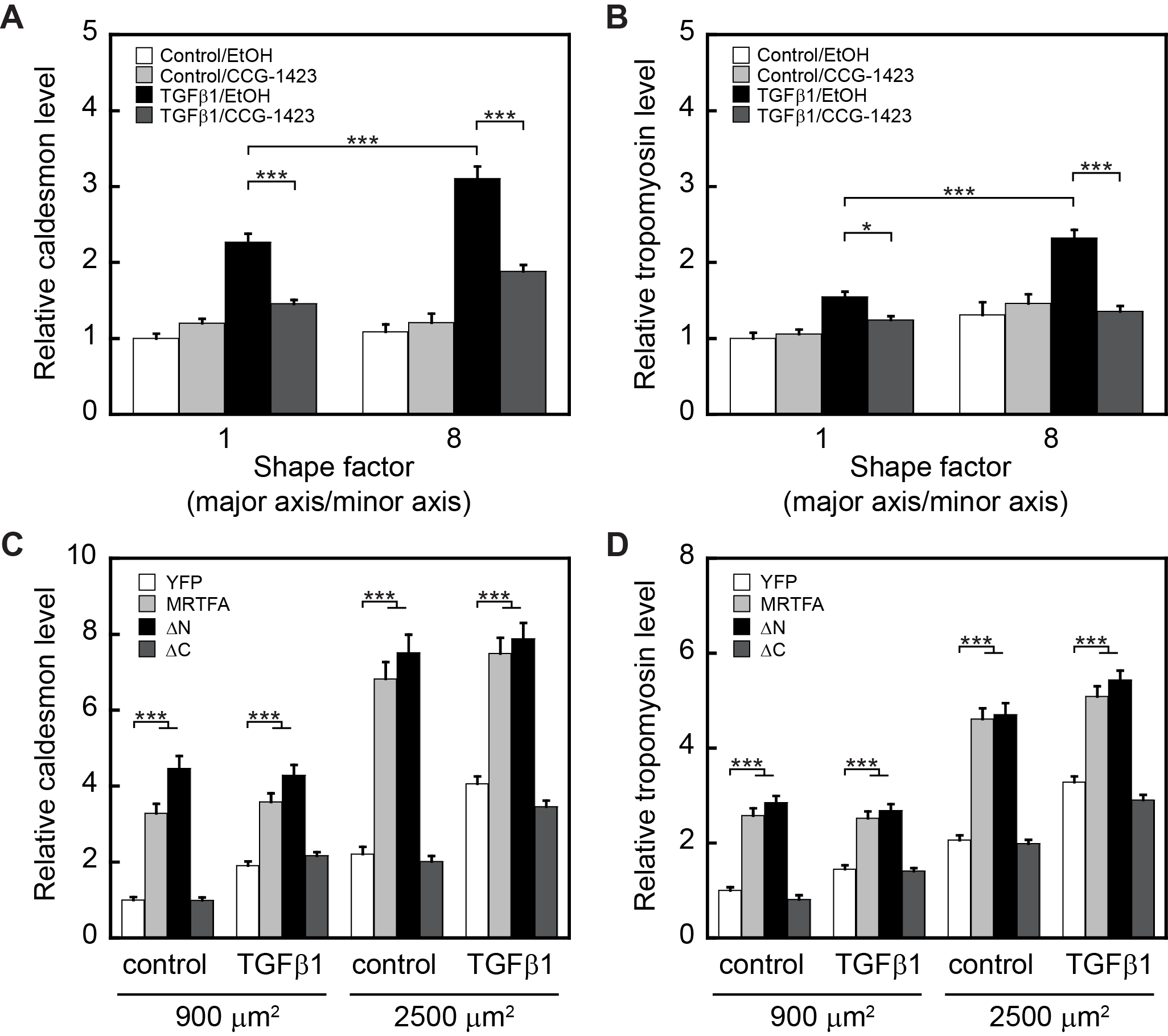

Supplement: Figure S4 — Myocardin-related transcription factor signaling controls cell shape-dependent expression of caldesmon and tropomyosin by TGFβ1. Quantification of the relative levels of (A) caldesmon and (B) tropomyosin after simultaneous treatment with TGFβ1 and ethanol vehicle or CCG-1423. Relative levels are computed in comparison to shape factor 1 control. *p < 0.05, ***p < 0.001. Overexpression of Flag-tagged MRTF-A and Flag-tagged MRTF-A-ΔN increase the expression of caldesmon and tropomyosin. Quantification of the relative levels of (C) caldesmon and (D) tropomyosin for YFP, MRTF-A, MRTF-A-ΔC, and MRTF-A-ΔN transfected NMuMG cells treated with TGFβ1 or control vehicle. Relative levels are computed in comparison to 900 μm2 control. ***p < 0.001 compared to YFP. (TIF) [file pone.0083188.s004.tif]
